# *catena*-Poly[[aqua­glycolatocopper(II)]-μ-chlorido]

**DOI:** 10.1107/S1600536808012166

**Published:** 2008-05-03

**Authors:** Hoong-Kun Fun, Jain John, Samuel Robinson Jebas, T Balasubramanian

**Affiliations:** a X-ray Crystallography Unit, School of Physics, Universiti Sains Malaysia, 11800 USM, Penang, Malaysia; b Department of Physics, National Institute of Technology, Tiruchirappalli 620 015, India

## Abstract

In the crystal structure of the title compound, [Cu(C_2_H_3_O_3_)Cl(H_2_O)]_*n*_, the Cu^II^ ion is five-coordinate in a distorted square-pyramidal geometry. Two O atoms from a chelating glycolate anion, an O atom from a coordinated water mol­ecule and a chloride anion comprise the basal plane. A chloride ion from a neighbouring unit occupies the apical position and these Cu—Cl—Cu bridges link the aqua­glycolatocopper(II) units into one-dimensional chains along the [001] direction. These chains are connected by O—H⋯O and O—H⋯Cl hydrogen bonds, forming an infinite three-dimensional polymeric network.

## Related literature

For background to the coordination chemistry of glycolic acid, see: Gao *et al.* (2004 ▶). For related structures, see: Dengel *et al.* (198 ▶7); Lanfranchi *et al.* (1993 ▶); Medina *et al.* (2000 ▶); Prout *et al.* (1993 ▶).
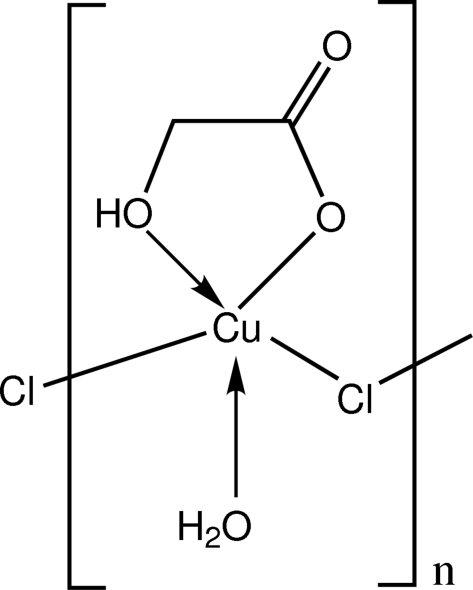


## Experimental

### 

#### Crystal data

[Cu(C_2_H_3_O_3_)Cl(H_2_O)]
*M* *_r_* = 192.05
Monoclinic, 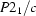

*a* = 7.6296 (2) Å
*b* = 10.0896 (3) Å
*c* = 7.4603 (2) Å
β = 109.632 (1)°
*V* = 540.91 (3) Å^3^
*Z* = 4
Mo *K*α radiation
μ = 4.45 mm^−1^
*T* = 100.0 (1) K
0.56 × 0.19 × 0.17 mm

#### Data collection

Bruker SMART APEXII CCD area-detector diffractometer
Absorption correction: multi-scan (*SADABS*; Bruker, 2005 ▶) *T*_min_ = 0.189, *T*_max_ = 0.512 (expected range = 0.174–0.470)
10874 measured reflections
2372 independent reflections
2147 reflections with *I* > 2σ(*I*)
*R*_int_ = 0.025

#### Refinement

*R*[*F* ^2^ > 2σ(*F* ^2^)] = 0.022
*wR*(*F* ^2^) = 0.058
*S* = 1.05
2372 reflections
93 parameters
All H-atom parameters refined
Δρ_max_ = 0.80 e Å^−3^
Δρ_min_ = −0.66 e Å^−3^

### 

Data collection: *APEX2* (Bruker, 2005 ▶); cell refinement: *APEX2*; data reduction: *SAINT* (Bruker, 2005 ▶); program(s) used to solve structure: *SHELXTL* (Sheldrick, 2008 ▶); program(s) used to refine structure: *SHELXTL*; molecular graphics: *SHELXTL*; software used to prepare material for publication: *SHELXTL* and *PLATON* (Spek, 2003 ▶).

## Supplementary Material

Crystal structure: contains datablocks global, I. DOI: 10.1107/S1600536808012166/sj2487sup1.cif

Structure factors: contains datablocks I. DOI: 10.1107/S1600536808012166/sj2487Isup2.hkl

Additional supplementary materials:  crystallographic information; 3D view; checkCIF report

## Figures and Tables

**Table 1:** Hydrogen-bond geometry (Å, °)

*D*—H⋯*A*	*D*—H	H⋯*A*	*D*⋯*A*	*D*—H⋯*A*
O1*W*—H1*W*1⋯Cl1^i^	0.76 (3)	2.32 (3)	3.0654 (10)	166 (2)
O1*W*—H2*W*1⋯O3^ii^	0.82 (2)	1.98 (2)	2.7400 (12)	153 (2)
O1—H1*O*1⋯O3^iii^	0.80 (2)	1.81 (2)	2.6086 (13)	177 (2)

Symmetry codes: (i) 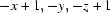; (ii) 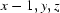; (iii) 
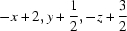
.

**Fig. 1.**
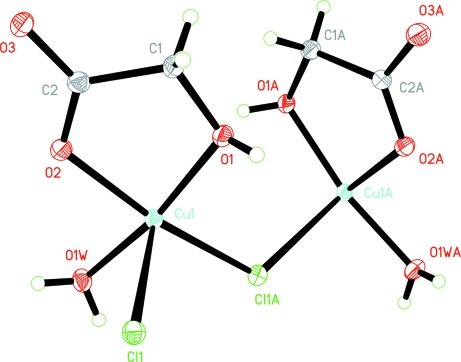
The molecular structure of the title compound, showing 50% probability displacement ellipsoids and the atomic numbering scheme. Symmetry code for atoms labelled A: x, -y + 1/2, z + 1/2.

**Fig. 2.**
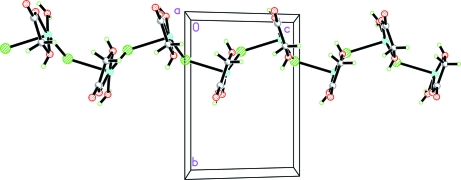
The crystal packing of the title compound, viewed along the a axis, showing a polymeric chain along the c axis.

## supplementary crystallographic information

### Comment

Glycolic acid (2-hydroxyethanoic acid) is a biologically active compound and has versatile binding modes for metals. (Gao *et al.*, 2004). A number of structures of metal complexes containing the glycolate ligand have been reported (Medina *et al.*, 2000; Prout *et al.*1993) with the chelating glycolate ligand coordinating to metal ions through the hydroxy and carboxy groups. In some coordination modes, the hydroxy groups of the glycolate are deprotonated (Dengel *et al.*,1987; Lanfranchi *et al.*, 1993). In this paper we report the structure of a novel three dimensional polymeric chloro-bridged copper complex with glycolate and water as auxiliary ligands.

In the asymmetric unit of the title compound, the Cu^II^ ion is five–coordinated with a distorted square–pyramidal geometry. The basal plane is formed by atoms O1 and O2 from the glycolate ligand in a chelating mode, a water oxygen and a chloride anion. Cl^-^ anions from neighbouring molecules link the [C_2_H_5_ClCuO_4_] units into polymeric chains along the [0 0 1] direction. The five membered ring [Cu1—O2—C2—C1—O1] is essentially planar with the maximum deviation from planarity being 0.008 (2)Å for the atom O1. The atom Cu1 is displaced by -0.1603 (1)Å out of the basal plane of the square pyramid towards atom Cl1.

The molecules are linked into one dimensional polymeric chains along the [0 0 1] direction through bridging chloride ions. Adjacent chains are interconnected by O—H···O, and O—H···Cl hydrogen bonds to form an infinite three dimensional polymeric network.

### Experimental

Equimolar amounts of glycolic acid and CuCl_2_ were dissolved in ethanol. The solution was refluxed at a temperature of 333°K for a period of 48 h. The clear blue colour solution was allowed to evaporate slowly yielding blue crystals of (I) after one month.

### Refinement

All the hydrogen atoms were located from the Fourier map and were allowed to refine freely.

### Crystal data

**Table:**

[Cu(C_2_H_3_O_3_)Cl(H_2_O)]	*F*_000_ = 380
*M**_r_* = 192.05	*D*_x_ = 2.358 Mg m^−^^3^
Monoclinic, *P*2_1_/*c*	Mo *K*α radiation λ = 0.71073 Å
Hall symbol: -P 2ybc	Cell parameters from 6364 reflections
*a* = 7.6296 (2) Å	θ = 2.8–41.4º
*b* = 10.0896 (3) Å	µ = 4.45 mm^−^^1^
*c* = 7.4603 (2) Å	*T* = 100.0 (1) K
β = 109.632 (1)º	Block, blue
*V* = 540.91 (3) Å^3^	0.56 × 0.19 × 0.17 mm
*Z* = 4	

### Data collection

**Table:**

Bruker SMART APEXII CCD area-detector diffractometer	2372 independent reflections
Radiation source: fine-focus sealed tube	2147 reflections with *I* > 2σ(*I*)
Monochromator: graphite	*R*_int_ = 0.025
*T* = 100.0(1) K	θ_max_ = 35.0º
φ and ω scans	θ_min_ = 2.8º
Absorption correction: multi-scan(SADABS; Bruker, 2005)	*h* = −12→12
*T*_min_ = 0.189, *T*_max_ = 0.512	*k* = −15→16
10874 measured reflections	*l* = −12→12

### Refinement

**Table:**

Refinement on *F*^2^	Secondary atom site location: difference Fourier map
Least-squares matrix: full	Hydrogen site location: inferred from neighbouring sites
*R*[*F*^2^ > 2σ(*F*^2^)] = 0.022	All H-atom parameters refined
*wR*(*F*^2^) = 0.058	*w* = 1/[σ^2^(*F*_o_^2^) + (0.035*P*)^2^ + 0.0909*P*] where *P* = (*F*_o_^2^ + 2*F*_c_^2^)/3
*S* = 1.05	(Δ/σ)_max_ < 0.001
2372 reflections	Δρ_max_ = 0.80 e Å^−^^3^
93 parameters	Δρ_min_ = −0.66 e Å^−^^3^
Primary atom site location: structure-invariant direct methods	Extinction correction: none

### Special details

**Table:**

Experimental. The data was collected with the Oxford Cyrosystem Cobra low-temperature attachment.
Geometry. All e.s.d.'s (except the e.s.d. in the dihedral angle between two l.s. planes) are estimated using the full covariance matrix. The cell e.s.d.'s are taken into account individually in the estimation of e.s.d.'s in distances, angles and torsion angles; correlations between e.s.d.'s in cell parameters are only used when they are defined by crystal symmetry. An approximate (isotropic) treatment of cell e.s.d.'s is used for estimating e.s.d.'s involving l.s. planes.
Refinement. Refinement of *F*^2^ against ALL reflections. The weighted *R*-factor *wR* and goodness of fit *S* are based on *F*^2^, conventional *R*-factors *R* are based on *F*, with *F* set to zero for negative *F*^2^. The threshold expression of *F*^2^ > σ(*F*^2^) is used only for calculating *R*-factors(gt) *etc*. and is not relevant to the choice of reflections for refinement. *R*-factors based on *F*^2^ are statistically about twice as large as those based on *F*, and *R*- factors based on ALL data will be even larger.

### Fractional atomic coordinates and isotropic or equivalent isotropic displacement parameters (Å^2^)

**Table:**

	*x*	*y*	*z*	*U*_iso_*/*U*_eq_	
Cu1	0.709943 (18)	0.143386 (14)	0.84591 (2)	0.01140 (5)	
Cl1	0.55544 (4)	0.22147 (3)	0.48054 (4)	0.01285 (6)	
O1	0.92698 (11)	0.26130 (9)	0.90217 (13)	0.01339 (15)	
O2	0.87233 (11)	0.01710 (9)	0.78289 (13)	0.01408 (15)	
O3	1.15672 (12)	−0.01023 (10)	0.76906 (14)	0.01787 (17)	
C1	1.08255 (15)	0.20052 (12)	0.86756 (17)	0.01349 (19)	
C2	1.03389 (15)	0.05935 (12)	0.80059 (16)	0.01302 (18)	
O1W	0.52646 (12)	0.00559 (10)	0.80898 (13)	0.01492 (16)	
H1A	1.114 (3)	0.2483 (19)	0.773 (3)	0.017 (4)*	
H1B	1.183 (3)	0.2001 (19)	0.981 (3)	0.014 (4)*	
H1W1	0.526 (3)	−0.050 (3)	0.740 (3)	0.033 (6)*	
H2W1	0.422 (3)	0.026 (2)	0.809 (3)	0.034 (6)*	
H1O1	0.904 (3)	0.331 (2)	0.849 (3)	0.025 (5)*	

### Atomic displacement parameters (Å^2^)

**Table:**

	*U*^11^	*U*^22^	*U*^33^	*U*^12^	*U*^13^	*U*^23^
Cu1	0.01055 (7)	0.00927 (8)	0.01456 (7)	−0.00050 (4)	0.00446 (5)	−0.00081 (4)
Cl1	0.01344 (10)	0.01174 (12)	0.01357 (10)	−0.00079 (9)	0.00481 (8)	0.00024 (8)
O1	0.0120 (3)	0.0100 (4)	0.0183 (4)	0.0004 (3)	0.0053 (3)	0.0003 (3)
O2	0.0115 (3)	0.0117 (4)	0.0188 (4)	−0.0006 (3)	0.0049 (3)	−0.0012 (3)
O3	0.0139 (3)	0.0142 (4)	0.0264 (4)	0.0003 (3)	0.0078 (3)	−0.0043 (3)
C1	0.0127 (4)	0.0117 (5)	0.0170 (4)	0.0001 (4)	0.0063 (4)	−0.0010 (4)
C2	0.0119 (4)	0.0122 (5)	0.0143 (4)	0.0003 (4)	0.0034 (3)	0.0009 (4)
O1W	0.0141 (3)	0.0129 (4)	0.0193 (4)	−0.0030 (3)	0.0076 (3)	−0.0037 (3)

### Geometric parameters (Å, °)

**Table:**

Cu1—O1W	1.9260 (9)	O2—C2	1.2686 (13)
Cu1—O2	1.9419 (8)	O3—C2	1.2548 (14)
Cu1—O1	1.9664 (9)	C1—C2	1.5138 (17)
Cu1—Cl1^i^	2.2480 (3)	C1—H1A	0.951 (19)
Cu1—Cl1	2.6983 (3)	C1—H1B	0.928 (19)
Cl1—Cu1^ii^	2.2479 (3)	O1W—H1W1	0.76 (3)
O1—C1	1.4344 (14)	O1W—H2W1	0.82 (2)
O1—H1O1	0.80 (2)		
			
O1W—Cu1—O2	89.08 (4)	C2—O2—Cu1	115.53 (8)
O1W—Cu1—O1	170.67 (4)	O1—C1—C2	109.61 (9)
O2—Cu1—O1	83.61 (4)	O1—C1—H1A	110.3 (11)
O1W—Cu1—Cl1^i^	92.11 (3)	C2—C1—H1A	109.0 (12)
O2—Cu1—Cl1^i^	168.29 (3)	O1—C1—H1B	108.4 (11)
O1—Cu1—Cl1^i^	93.90 (3)	C2—C1—H1B	109.3 (12)
O1W—Cu1—Cl1	90.94 (3)	H1A—C1—H1B	110.2 (16)
O2—Cu1—Cl1	92.56 (3)	O3—C2—O2	123.59 (11)
O1—Cu1—Cl1	95.15 (3)	O3—C2—C1	118.20 (10)
Cl1^i^—Cu1—Cl1	99.065 (9)	O2—C2—C1	118.20 (10)
Cu1^ii^—Cl1—Cu1	120.780 (12)	Cu1—O1W—H1W1	118.1 (17)
C1—O1—Cu1	113.04 (7)	Cu1—O1W—H2W1	118.3 (16)
C1—O1—H1O1	109.9 (16)	H1W1—O1W—H2W1	114 (2)
Cu1—O1—H1O1	113.7 (16)		
			
O1W—Cu1—Cl1—Cu1^ii^	−165.23 (3)	O1—Cu1—O2—C2	−0.62 (8)
O2—Cu1—Cl1—Cu1^ii^	−76.11 (3)	Cl1^i^—Cu1—O2—C2	−78.90 (16)
O1—Cu1—Cl1—Cu1^ii^	7.70 (3)	Cl1—Cu1—O2—C2	94.27 (8)
Cl1^i^—Cu1—Cl1—Cu1^ii^	102.489 (19)	Cu1—O1—C1—C2	−1.27 (11)
O1W—Cu1—O1—C1	39.6 (3)	Cu1—O2—C2—O3	178.65 (9)
O2—Cu1—O1—C1	1.08 (8)	Cu1—O2—C2—C1	0.04 (13)
Cl1^i^—Cu1—O1—C1	169.58 (7)	O1—C1—C2—O3	−177.86 (10)
Cl1—Cu1—O1—C1	−90.94 (7)	O1—C1—C2—O2	0.83 (15)
O1W—Cu1—O2—C2	−174.83 (8)		

Symmetry codes: (i) *x*, −*y*+1/2, *z*+1/2; (ii) *x*, −*y*+1/2, *z*−1/2.

### Hydrogen-bond geometry (Å, °)

**Table:**

*D*—H···*A*	*D*—H	H···*A*	*D*···*A*	*D*—H···*A*
O1W—H1W1···Cl1^iii^	0.76 (3)	2.32 (3)	3.0654 (10)	166 (2)
O1W—H2W1···O3^iv^	0.82 (2)	1.98 (2)	2.7400 (12)	153 (2)
O1—H1O1···O3^v^	0.80 (2)	1.81 (2)	2.6086 (13)	177 (2)

Symmetry codes: (iii) −*x*+1, −*y*, −*z*+1; (iv) *x*−1, *y*, *z*; (v) −*x*+2, *y*+1/2, −*z*+3/2.
